# Autonomic Nervous System Regulation of Epicardial Adipose Tissue: Potential Roles for Regulator of G Protein Signaling-4

**DOI:** 10.3390/cimb44120415

**Published:** 2022-12-05

**Authors:** Alexandra M. Carbone, Giselle Del Calvo, Deepika Nagliya, Karina Sharma, Anastasios Lymperopoulos

**Affiliations:** Laboratory for the Study of Neurohormonal Control of the Circulation, Department of Pharmaceutical Sciences, Nova Southeastern University College of Pharmacy, Ft. Lauderdale, FL 33328-2018, USA

**Keywords:** autonomic nervous system, adrenergic receptors, atrial fibrillation, epicardial adipose tissue, heart failure, muscarinic receptors, regulator of g protein signaling-4, signal transduction

## Abstract

The epicardial adipose tissue (EAT) or epicardial fat is a visceral fat depot in the heart that contains intrinsic adrenergic and cholinergic nerves, through which it interacts with the cardiac sympathetic (adrenergic) and parasympathetic (cholinergic) nervous systems. These EAT nerves represent a significant source of several adipokines and other bioactive molecules, including norepinephrine, epinephrine, and free fatty acids. The production of these molecules is biologically relevant for the heart, since abnormalities in EAT secretion are implicated in the development of pathological conditions, including coronary atherosclerosis, atrial fibrillation, and heart failure. Sympathetic hyperactivity and parasympathetic (cholinergic) derangement are associated with EAT dysfunction, leading to a variety of adverse cardiac conditions, such as heart failure, diastolic dysfunction, atrial fibrillation, etc.; therefore, several studies have focused on exploring the autonomic regulation of EAT as it pertains to heart disease pathogenesis and progression. In addition, Regulator of G protein Signaling (RGS)-4 is a protein with significant regulatory roles in both adrenergic and muscarinic receptor signaling in the heart. In this review, we provide an overview of the autonomic regulation of EAT, with a specific focus on cardiac RGS4 and the potential roles this protein plays in this regulation.

## 1. Introduction

Epicardial adipose tissue (EAT) is the fat deposit surrounding the heart, located between the myocardium and the visceral pericardium [1]. At the molecular level, fat exists as lipid in the form of triglycerides [1]. While body fat is mostly found in adipose tissue, it also exists within other tissues. EAT, contrary to paracardial fat, separated from the myocardium by the pericardium, has no physical boundaries with the underlying myocardium [1]. EAT is perfused by the coronary arteries and serves to store energy in the form of lipids for the myocardium, for thermoregulation, to protect autonomic ganglia and neuronal tissue, and regulation of coronary artery vasomotion and luminal size [1]. A range of pathophysiologic mechanisms could contribute to an association between EAT and atrial fibrillation (AFib) [2]. EAT can lead to Afib via structural and electrical remodeling of the atria by both direct (infiltration of adipose tissue leading to altered atrial electrophysiological properties) and indirect mechanisms (secretion of paracrine modulators of myocardial inflammation and oxidative stress) [2]. 

## 2. Sympathetic Nervous System (SNS) of the Heart

The SNS is responsible for orchestrating the body’s response to situations of stress or emergency (“fight-or-flight”) and communicates with the myocardium directly via cardiac ganglia along the visceral column (paravertebral ganglia) [3]. The (postganglionic) sympathetic neurotransmitter is norepinephrine (NE), although the neurotransmitter of the preganglionic neurons of both the sympathetic and parasympathetic systems is acetylcholine (Ach) [3]. Thus, these sympathetic postganglionic fibers are called (nor)adrenergic neurons. NE and its close relative epinephrine (Epi), a hormone secreted by the adrenal medulla, exert their actions through three α_1_, three α_2_, and three β adrenergic receptor (AR) subtypes, which are all G protein-coupled receptors (GPCRs) [4,5,6,7,8,9,10,11,12,13,14,15]. β_1_ARs are expressed in the heart (in the sinoatrial and atrioventricular nodes, and in atrial and ventricular cardiomyocytes). Their activation increases heart rate (positive chronotropy), contractility (positive inotropy), and atrioventricular node conduction velocity (positive dromotropy) [4,5,6,7,8,9,10,11,12,13,14,15]. β_1_AR is also present in the juxtaglomerular apparatus cells of the kidney where it induces renin release to activate the renin–angiotensin–aldosterone system (RAAS). β_2_ARs are mainly expressed in vascular smooth muscle, skeletal muscle, and in the coronary circulation [6]. Their activation elicits vasodilatation, which, in turn, increases blood perfusion to target organs. These receptors reside in non-sympathetic innervated tissues, so they are primarily stimulated by circulating Epi [6]. In addition, β_2_ARs display much higher affinity for Epi than for NE [7]. There are also some low numbers of expression of β_2_ARs in cardiomyocytes [16]. α_1_ARs are expressed in vascular smooth muscle proximal to sympathetic nerve terminals and they mediate vasoconstriction [16,17,18]. Cardiac myocytes also express some (minute) levels of α_1_ARs [16]. Finally, α_2_ARs are expressed in vascular smooth muscle distal from sympathetic nerve terminals, where they also elicit vasoconstriction, but they are also in the central nervous system mediating autoinhibition of sympathetic outflow, and in the adrenal medulla mediating auto-inhibition of NE and Epi secretion [19,20,21,22,23]. Myocardial contractility represents the ability of the heart to increase force of contraction, determined by the strength of the actomyosin filament interaction, which, in turn, depends on the cytoplasmic Ca^2+^ concentration of the myocyte [23]. Catecholamine binding to the β_1_AR is among the most powerful stimuli for elevation of intracellular Ca^2+^ concentration in the cardiomyocyte, and, consequently, for contraction of both the atria and ventricles [10,24]. Of note, the β_3_AR subtype mediates atrial contraction, but ventricular relaxation rather than contraction, via nitric oxide generation in the myocardium [25,26].

## 3. Parasympathetic Nervous System (PNS) of the Heart

The PNS plays, in most (but not all) cases, an antagonistic, to the sympathetic system, role in regulating heart function [3]. Parasympathetic preganglionic fibers innervate organs of the thorax and upper abdomen as parts of the vagus nerve, which carries ~75% of all parasympathetic nerve fibers passing to the heart and many other visceral organs [3]. The preganglionic fibers synapse within the ganglion, and then short postganglionic fibers leave the ganglia to the target organ. Thus, in the parasympathetic system, preganglionic neurons are generally longer than postganglionic neurons [3]. ACh is the neurotransmitter of both preganglionic and postganglionic parasympathetic neurons (thus, they are called cholinergic neurons). ACh exerts its effects via two types of cholinergic receptors called nicotinic receptors (nAChRs) and muscarinic receptors (mAChRs) [27]. mAChRs are GPCRs located in the membranes of effector cells at the end of postganglionic parasympathetic nerves and at the ends of cholinergic sympathetic fibers. Responses from these receptors are excitatory and relatively slow [3,11]. The nAChRs are ligand-gated ion channels located at synapses between pre- and postganglionic neurons of the sympathetic and parasympathetic pathways. Exactly because they are ion channels, nAChRs produce rapid, excitatory responses, in contrast to mAChRs [11]. Out of the five different known subtypes of mAChRs, the M_2_ mAChR is the major cholinergic receptor subtype in the mammalian heart. It is abundantly expressed in the atria and in conductive fibers, such as the sino-atrial and atrio-ventricular nodes, but, notably, its expression is negligible in the ventricles [28,29,30]. This means that ACh reduces heart rate via this receptor, shortening both action potential duration and conduction velocity (negative chronotropy and dromotropy) [31,32,33]. Regarding contractility, however, ACh appears capable of directly exerting negative inotropy only in human atrial myocytes, whereas, in human ventricular myocytes, it merely blocks the positive inotropic action of the catecholamines, i.e., indirect inhibition of contractility [4], which probably reflects the more dense parasympathetic innervation of the human atria and nodal regions vs. ventricles. The result is that the effect of PNS on overall cardiac contractility is minimal, at least in humans [4,33]. M_3_ receptors are mainly expressed in vascular endothelium, where they mediate nitric oxide-dependent vasodilatation [3]. In conclusion, the parasympathetic system opposes the effects of the sympathetic nervous system on heart rate and nodal conduction but the effect on myocardial contractility is minimal. Nevertheless, reduced ACh secretion due to decreased neuronal cholinergic activity has been documented to accompany various cardiovascular diseases, such as arrhythmias, hypertension, myocardial infarction, and heart failure [34,35,36,37,38,39,40,41,42]. 

## 4. Autonomic Dysregulation and EAT: Implications for Human AFib and Heart Failure

**Figure 1:**
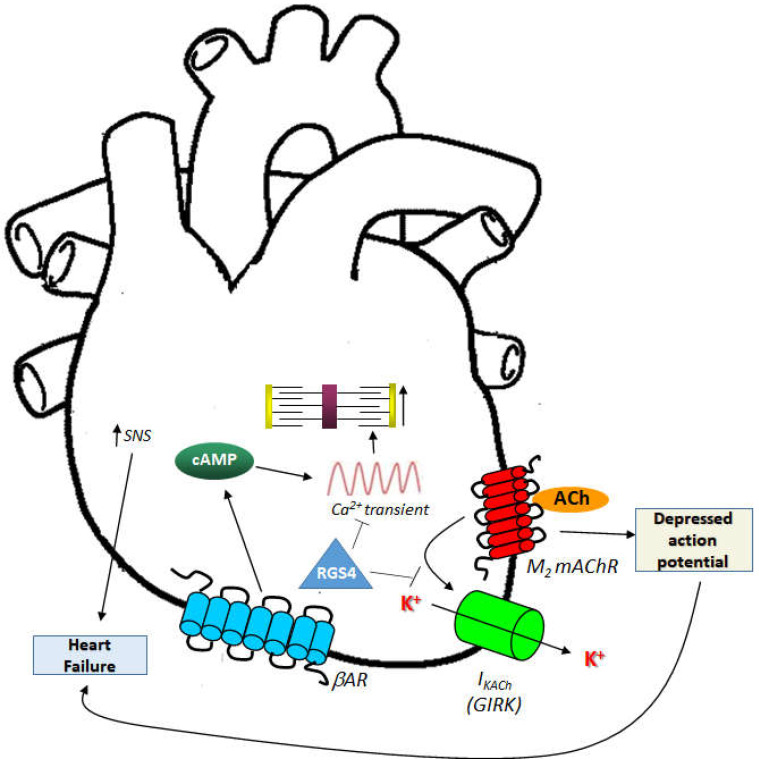
Important mechanisms underlying cardiac autonomic dysfunction contributing to heart failure or AFib pathogenesis, and possible roles of RGS4 in them. SNS, Sympathetic nervous system; GIRK, G protein-gated (coupled) inwardly rectifying K^+^ channel; cAMP, Cyclic 3′,5′-adenosine monophosphate; ACh, Acetylcholine; mAChR: Muscarinic cholinergic receptor; AR, Adrenergic receptor; I_KACh_, Cholinergic potassium (hyperpolarizing) current. See text for details.

**Figure 2:**
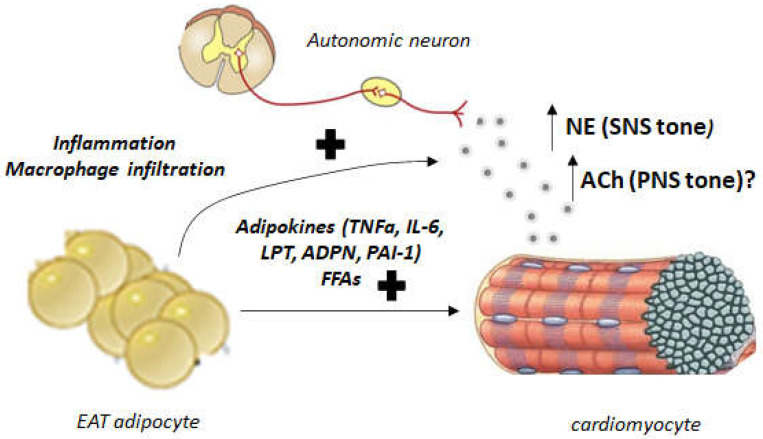
Pathophysiological interplay between EAT cells, autonomic neurons, and the myocardium. EAT dysfunction, caused e.g., by inflammation, increases SNS tone (NE release from sympathetic neurons), and, perhaps, PNS tone (ACh release) within the heart, through elevated secretion of adipokines and FFAs. This leads to sympathetic hyperactivity and autonomic dysfunction of the myocardial cells, contributing to heart failure, AFib pathogenesis, coronary heart disease, and other myocardial maladies. Of note, the very same adipokines and FFAs, whose secretion from EAT is increased, can also directly (i.e., independently of autonomic nerve activation) affect the cardiomyocytes, inducing apoptosis, inflammation, and other maladaptive processes that also contribute to heart failure progression. NE, Norepinephrine; SNS, Sympathetic nervous system; ACh, Acetylcholine; PNS, Parasympathetic nervous system; TNFα, Tumor necrosis factor-α; IL-6, Interleukin-6; LPT, Leptin; ADPN, Adiponectin; PAI-1, Plasminogen activator inhibitor-1; FFAs, Free fatty acids (saturated). See also text.

The autonomic nervous system has ganglions within the heart located in the EAT pads that regulate cardiac autonomic nervous input [43]. Vagal stimulation is modulated through multiple cardiac ganglionic plexi prior to arriving at the sinoatrial and atrioventricular nodes [43,44]. The autonomic dysfunction leading to AFib is well documented. The cholinergic system contributes significantly to AFib in young, otherwise healthy patients [45]. Significant PNS innervation of the atrial muscle exists that extends into the pulmonary circulation [46]. As mentioned above, ACh-activated mAChRs (particularly of the M_2_ subtype, which is G_i/o_ protein-coupled GPCR) stimulate G protein-gated atrial K^+^ channels (GIRKs) leading to hyperpolarization (cholinergic potassium hyperpolarizing current, I_KACh_) (Figure 1) [47]. Through activation of the adenylyl cyclase-inhibitory Gαi subunits, M_2_ mAChR also inhibits synthesis and signaling of the second messenger cyclic 3′,5′-adenosine monophosphate (cAMP), produced by the activation of βARs of the cardiac SNS (Figure 1) [24,47,48]. This results in shortening of the atrial action potential duration with increased spatial heterogeneity [48] allowing for AFib occurrence (Figure 1). On the other hand, the SNS can also trigger AFib or ventricular arrhythmias by directly eliciting intracellular Ca^2+^ elevations in response to βAR activation [24,49,50] (Figure 1). Since autonomic ganglionic plexi are found within the EAT, EAT plays important roles in regulating autonomic nervous system tone (Figure 2). Indeed, in obese or diabetic individuals, autonomic signals emanating from EAT are dysregulated and cause arrhythmias [51]. Abnormal increase in pericardial or epicardial fat is associated with abnormal regulation of autonomic nervous system activity, which might lead to increased ventricular arrhythmias and enhanced morbidity and mortality [51]. 

Since the autonomic nervous system crucially regulates heart rhythm and ganglionated plexi are located in EAT (Figure 2) [52,53], activation of these plexi can cause both parasympathetic and sympathetic stimulation, resulting in shortened action potentials and increased calcium transients, respectively (Figure 1) [44]. EAT can influence these encased ganglionated plexi contributing to arrhythmogenesis. Indeed, botulinum toxin injection inhibits ACh release from preganglionic nerve terminals into epicardial fat pads and reduces cardiac autonomic nervous activity and AFib by potentially suppressing ganglionated plexi [54,55]. In biopsies, explants, or primary cultures obtained from the EAT of 85 patients that underwent open-heart surgery, M_3_ mAChR (a G_q/11_ protein GPCR that induces calcium signaling) was found upregulated after adipogenesis induction and cholinergic fibers in EAT were detected by vesicular ACh transporter levels and acetylcholinesterase activity [55]. ACh treatment modified the secretome of the EAT of these patients, with various EAT-secreted proteins displaying differential levels between patients who developed AFib post-surgery compared to those who did not [56]. Thus, cholinergic activity of EAT regulates the interplay among EAT, autonomic nervous system dysfunction, and AFib in a clinically meaningful manner [57]. Another study examined the relationship between vagal response during cryoballoon catheter ablation for AFib and cardiac autonomic nervous system modulation by evaluating EAT locations and heart rate variability analysis [57]. Vagal effects on the cardiac autonomic nervous system in patients with paroxysmal AFib who underwent second-generation cryo-balloon ablation were compared between patients receiving vagal stimulation and patients that did not. The vagal response-receiving group exhibited greater EAT volume encasing the left atrium-left superior pulmonary vein junction than the non-vagal stimulated group [57]. Additionally, volume of the EAT occupying this anatomical location correlated well with changes in heart rate variability immediately post-cryoablation [57]. Thus, EAT volume on top of the left atrium–left superior pulmonary vein junction is useful for heart rate variability assessment and autonomic nervous system modulation of the heart [57]. A similar study from a Turkish group demonstrated that patients with higher EAT volume displayed significantly more heart rate variability and turbulence compared to patients with lower volume EAT [58]. The authors concluded that autonomic imbalance is directly related to EAT thickness and, thus, EAT volume and composition may play an important arrhythmogenic role, not necessarily limited to Afib [58]. Further support for this was provided by a study in Japanese obese subjects, which performed a cross-sectional analysis of their EAT thickness [59]. These authors found that higher EAT thickness correlated with impaired recovery and lower cardiorespiratory fitness compared to subjects with lower EAT thickness. Moreover, higher EAT thickness in men was reported to represent cardiac autonomic dysfunction and poor parasympathetic response to exercise [59,60].

Of note, a neural pathway from the cervical vagus trunk to the sinoatrial node and left atrium has been suggested to run through the sinoatrial node-encasing EAT but to eventually converge at the atrioventricular node-encasing EAT, thus serving as an “integration center” for the former EAT in modulation of sinoatrial node function [61]. In other words, the atrioventricular node-encasing EAT may play a more critical role in the initiation and maintenance of AFib. However, a study in patients undergoing coronary artery bypass grafting (CABG) surgery showed that, although maintenance of the EAT prevented attenuation of parasympathetic tone after CABG, it did not reduce post-surgery AFib or total hospital costs in any appreciable way [62]. As far as EAT involvement in diabetic heart abnormalities is concerned, a study examined EAT metabolism in heart failure patients with or without diabetes and found that the glucose uptake differential between basal and insulin stimulation was significantly depressed in epicardial vs. control, subcutaneous adipocytes [63]. Moreover, lipolysis induced by isoproterenol, a βAR full agonist, was lower in EAT than in subcutaneous fat, correlating well with lipolysis, lipid storage, and inflammation-related gene expression [63]. Finally, fatty acid composition of both fat tissues was significantly altered by diabetes. In conclusion, significant metabolic differences exist between EAT and subcutaneous adipose tissue in diabetic heart failure and EAT metabolism could be targeted therapeutically for diabetic heart failure treatment [64].

Finally, an Italian study in systolic heart failure patients identified a highly significant correlation between EAT thickness and the extent of cardiac sympathetic denervation [65]. Specifically, EAT thickness was reported to be useful as an independent predictor of SNS dysfunction, since left ventricular mass, EAT thickness, and cardiac sympathetic denervation were found to correlate well with one another in systolic heart failure patients [66]. EAT becomes thicker as cardiac SNS activity decreases and left ventricular mass increases. In addition, this study demonstrated that EAT is a source of catecholamines itself, as both NE and Epi were present in higher concentrations in EAT compared with subcutaneous adipose tissue [66]. In heart failure patients, NE levels were increased 5.6-fold in EAT compared with subcutaneous adipose tissue and 2-fold compared with plasma [66]. Importantly, these increases were attributed to increased catecholamine biosynthesis within the EAT, since the catecholamine biosynthetic enzymes tyrosine hydroxylase and dopamine beta-hydroxylase were found massively upregulated at both the mRNA and protein levels, compared to the control, subcutaneous adipose tissue of the patients [66]. Although the reported elevations in expression of these enzymes were astonishingly huge (~8-fold for the mRNAs and ~15-fold (!) for the proteins), raising concerns about the accuracy of the reported values, this study clearly identified human EAT as a significant source of both NE and Epi, at least in the context of systolic heart failure, which might contribute to the well-documented SNS hyperactivity that accompanies and aggravates human heart failure [65,66]. The increased catecholamine biosynthetic activity of EAT in systolic heart failure, which is obviously the result of a thickened EAT (higher volume EAT contains more adipocytes synthesizing more catecholamines), adds to the total catecholamine accumulation in the failing heart’s EAT [65]. In conclusion, this study provides evidence for EAT thickness being an index of cardiac SNS activity and derangement and for use in determining prognosis in systolic heart failure patients. However, whether these findings apply also to diastolic heart failure or HFpEF (heart failure with preserved ejection fraction) patients [67,68,69] remains an open question.

## 5. RGS4 and EAT Regulation

Regulator of G protein Signaling (RGS)-4 is highly expressed in the heart and brain [70,71,72,73,74]. This protein belongs to the B/R4 group of RGS proteins and inactivates Gi/o- and G_q/11_ protein signaling by effectively serving as guanosine triphosphatase activating protein (GAP) for the alpha subunits of these G proteins [70,71,72,73,74,75]. Importantly, RGS4 has been reported, uniquely among RGS proteins, to directly bind Gi/o-derived free G_βγ_ subunits and phospholipase C (PLC)-β, thereby blocking PLCβ activation and downstream calcium signaling independently of its GAP action on the Gα subunits [76,77]. RGS4 is abundantly expressed in the sinoatrial and atrioventricular nodal regions of the heart, as well as throughout the atrial muscle [78,79]. Exogenous overexpression of RGS4 in cardiomyocytes attenuates endothelin receptor signaling, reducing PLCβ activation, contractility in the long term, and cardiac hypertrophy [80,81,82]. Indeed, RGS4 ameliorates cardiac hypertrophy induced by pressure overload via direct inhibition of the Gq protein-dependent pro-hypertrophic signaling in murine hearts [80,81,82]. RGS4 is upregulated in rat hypertrophic hearts [83] and, importantly, in human failing hearts from both acute and end stage chronic heart failure patients [84,85]. Moreover, RGS4 protects against abnormal calcium transients and signaling that leads to tachyarrhythmias/AFib (Figure 1) [86]. 

At the same time, RGS4 is essential for the cholinergic regulation of heart rate through the M_2_ mAChR [78,87,88]. Indeed, RGS4 (and its homolog RGS6) is required for desensitization and rapid deactivation, as well as normal activation, of M_2_ mAChR-mediated I_KACh_, as it inactivates M_2_ mAChR-induced G_i/o_ protein signaling that operates this current (G_i/o_ protein-derived free Gβγ opens the G protein-gated inwardly rectifying K^+^ channels (GIRKs) responsible for I_KACh_) (Figure 1) [78,87,88]. Thus, RGS4 may protect not only against calcium signaling-induced tachyarrhythmias and AFib, but also against cholinergic-induced bradycardia (Figure 1).

Finally, we recently reported on the crucial role of RGS4 in regulation of the free fatty acid receptor (FFAR)3, also known as GPR41 [89]. FFAR3 is a GPCR activated by short chain fatty acids, e.g., propionate, butyrate, and regulates cardiovascular function via effects in peripheral sympathetic neurons, wherein it promotes neuronal firing and NE synthesis/release [90]. RGS4 was found to inactivate cardiac FFAR3 G_i/o_ protein signaling, resulting in cardioprotection against short chain fatty acid-dependent pro-inflammatory and pro-fibrotic effects [89]. In addition, cardiac βARs stimulate RGS4 to impede this FFAR3 signaling [89]. Importantly, RGS4 also opposed FFAR3-dependent NE release from sympathetic neurons co-cultured with cardiac myocytes, thereby preserving cardiac βAR function [89]. This provides another line of evidence for the cardioprotective role of RGS4 against inflammation and fibrosis, two maladaptive processes of the heart known to lead to AFib, arrhythmias, and heart failure [91,92]. 

Based on all the above, it is tempting to speculate that RGS4 may be involved in the autonomic regulation of EAT, as it is clearly (and in a very essential manner) involved in the autonomic regulation of the myocardium. Investigations of RGS4 expression levels in human EAT and of potential alterations in epicardial fat RGS4 levels in heart disease (e.g., heart failure, AFib) are certainly worth pursuing, since RGS4 levels may very well correlate (in an inverse proportional manner) with levels of SNS and PNS activities in EAT of cardiovascular patients. 

## 6. Conclusions

Although our understanding of the relationship between EAT and AFib or heart failure has increased dramatically in recent years, this exciting new field of research is still in its infancy. An increasing number of clinical and epidemiological studies demonstrate consistent associations between epicardial fat and AFib, but more research is needed to establish causation. Additional evidence from larger, prospective cohort studies is imperative to draw statistically meaningful comparisons of the different visceral adipose tissues and sub-depots of epicardial fat. Both basic science and translational studies are needed to enhance our understanding of the mechanisms underlying the role of EAT in the autonomic dysfunction that precipitates AFib and heart failure. Identification and validation of novel molecular targets, such as RGS4, whose role in autonomic regulation of the myocardium and of EAT is only beginning to unravel, will be key to obtaining the full picture of EAT’s role in cardiac physiology and disease and how to exploit this fat deposit for therapeutic purposes. Obesity will continue to emerge as a principal risk factor and causative trigger of both AFib and heart failure, but also of other cardiovascular diseases in the coming years. Thus, investigations into the roles the various human body fat depots, including EAT, play in the pathophysiology of AFib and heart failure will certainly continue to be one of the hottest research areas in biomedicine. The race to come up with new weapons to equip the cardiologist of the future to combat heart disease is only bound to intensify.

## Data Availability

Not applicable.
